# Safety monitoring experience of single-low dose primaquine co-administered with artemether–lumefantrine among providers and patients in routine healthcare practice: a qualitative study in Eastern Tanzania

**DOI:** 10.1186/s12936-021-03921-w

**Published:** 2021-10-09

**Authors:** Dominic Mosha, Mwaka A. Kakolwa, Muhidin K. Mahende, Honorati Masanja, Salim Abdulla, Chris Drakeley, Roland Gosling, Joyce Wamoyi

**Affiliations:** 1 Ifakara Health Institute, Plot 463, Kiko Ave, Mikocheni, P.O Box 78373, Dar es Salaam, Tanzania; 2 Department of Immunology and Infection, London School of Hygiene & Tropical Medicine, London, UK; 3 Department of Epidemiology and Biostatistics, University of California San Francisco, San Francisco, CA USA; 4 Global Health Group, Malaria Elimination Initiative, University of California, San Francisco, CA USA; 5 National Institute for Medical Research, Mwanza, Tanzania

**Keywords:** Malaria, Primaquine, Artemether–lumefantrine, Safety, Monitoring

## Abstract

**Background:**

Primaquine is a gametocytocidal drug recommended by the World Health Organization (WHO) in a single-low dose combined with artemisinin-based combination therapy (ACT) for the treatment and prevention of *Plasmodium falciparum* malaria transmission. Safety monitoring concerns and the lack of a universal validated and approved primaquine pharmacovigilance tool is a challenge for a national rollout in many countries. This study aimed to explore the acceptance, reliability and perceived effectiveness of the primaquine roll out monitoring pharmacovigilance tool (PROMPT).

**Methods:**

This study was conducted in three dispensaries in the Coastal region of Eastern Tanzania. The study held six in-depth interviews with healthcare providers and six participatory focus group discussions with malaria patients (3) and parents/guardians of sick children (3). Participants were purposively sampled. Thematic analysis was conducted with the aid of NVivo qualitative analysis software.

**Results:**

The respondents’ general acceptance and perceived effectiveness of the single-low dose primaquine and PROMPT was good. Screening procedure for treatment eligibility and explaining to patients about the possible adverse events was considered very useful for safety reasons. Crushing and dissolving of primaquine tablet to get the appropriate dose, particularly in children, was reported by all providers to be challenging. Transport costs and poor access to the health facility were the main reasons for a patient failing to return to the clinic for a scheduled follow-up visit. Treatment was perceived to be safe by both providers and patients and reported no case of a severe adverse event. Some providers were concerned with the haemoglobin drop observed on day 7.

**Conclusion:**

Single-low dose primaquine was perceived to be safe and acceptable among providers and patients. PROMPT demonstrated to be a reliable and user-friendly tool among providers. Further validation of the tool by involving the National Malaria Control Programme is pivotal to addressing key challenges and facilitating primaquine adoption in the national policy.

## Background

About 3.2 billion people are at risk of malaria despite the progressive global decline of malaria prevalence. In 2019, there were over 200 million new cases of malaria and 409,000 deaths; approximately 94% occurred in sub-Saharan Africa [1]. Malaria is the leading cause of morbidity and mortality in Tanzania, especially in children under five and pregnant women. Each year, about 12 million people contract malaria in Tanzania and approximately 80,000 deaths [2].

Global efforts for malaria control, including the use of insecticide-treated nets (ITNs), malaria rapid diagnostic test (RDT), indoor residual spraying (IRS), and effective chemotherapy, have successfully managed to reduce malaria by two-fold in the past 15 years [3]. To achieve the World Health Organization (WHO) goal of eliminating malaria by 2035, there is a need to address potential challenges facing artemisinin-based combination therapy (ACT), particularly with emerging of *P. falciparum* resistance against artemisinins and their poor gametocytocidal effect [4]. The use of effective chemotherapy, such as primaquine with gametocytocidal properties may reduce *P. falciparum* transmission and suppress the spread of artemisinin-resistant falciparum malaria [5]. Thus, the WHO currently recommends a single low-dose (SLD) of primaquine (0.25 mg base/kg) to be administered to all patients with parasitological-confirmed *P. falciparum* malaria on the 1st day of treatment in addition to ACT, except for pregnant women and infants < 6 months [6]. The strategy is likely to be effective in reducing malaria transmission in areas of low-intensity malaria transmission. Testing for glucose-6-phosphate dehydrogenase (G6PD) deficiency is not required with the recommended low primaquine dose [6].

The main concern about the safety of primaquine is the risk for acute haemolytic anaemia (AHA) in G6PD deficient individuals. There is strong evidence that supports the safety of SLD primaquine, even in people with G6PD deficiency by not causing clinically significant AHA [7–10]. Despite the latter, many sub-Saharan countries still hesitate to include primaquine in their national malaria treatment policies due to challenges related to safety monitoring [11]. Screening for AHA life-threatening risk factors associated with primaquine, such as low haemoglobin level and previous history of aminoquinoline drug reaction may be challenging in routine practice. This challenge is even more prominent in remote facilities in many developing countries where medical resources including, qualified clinicians and supplies are scarce [12]. This is a paramount concern considering little is known about pharmacovigilance tools that may be useful in a real-world environment and existing uncertainties around practical implementation of the best clinical management of haemolytic reaction.

However, the deployment option of SLD primaquine is still widely accepted in the treatment of confirmed clinical malaria cases, among other interventions [13]. It is, therefore, essential for the National Malaria Control Programme (NMCP) to have an effective and feasible monitoring mechanism in place for evaluating the effectiveness and safety of malaria case management during the rollout of SLD primaquine. Having an effective and feasible primaquine pharmacovigilance tool may fast track resource-limited nations to adopt primaquine in their national policy as a treatment of choice for uncomplicated *P. falciparum*.

The Malaria Elimination Initiative (MEI) at the University of California, San Francisco (UCSF) has developed a comprehensive ‘Primaquine *roll out monitoring pharmacovigilance tool*’ (PROMPT) [14], which serves as a healthcare provider guide for monitoring this new malaria treatment regimen from day 0 to 7. The tool was used to support NMCP policy adoption and roll out of SLD primaquine in Eswatini (formerly Swaziland) and Senegal [15]. There is a need to validate the tool in East African countries, where there is a significant shortage of qualified clinicians, scarce medical resources and supplies, a low literacy rate in the community and a relatively high G6PD deficiency [16]. Therefore, the PROMPT was tested in a real-world environment at the lowest health facility delivery level in a rural coastal area in Eastern Tanzania to demonstrate its acceptance, reliability, and perceived effectiveness among health care providers in managing uncomplicated malaria cases with artemether–lumefantrine (AL) + SLD primaquine.

## Methods

### Study setting

The study was conducted in Kibiti and Ikwiriri districts in the Coastal region, Eastern Tanzania. The two districts border each other and have similar geographical, economic and population characteristics. The estimated total population in the two districts is 248,230 in 94 registered villages and 385 hamlets. The main economic activities in the area are small-scale farming, fishing, retailing and animal keeping. The districts are part of the hot and humid coastal plain with varying tropical climatic conditions. Malaria transmission in the area is hyperendemic, highest during and after the long rain period. Malaria is one of the main diseases responsible for most outpatient health facility attendance in the district. The two districts have two hospitals, five health centres and 48 dispensaries predominantly owned by the government. The study area has been described in detail elsewhere [17].

### PROMPT trial description

The trial was conducted from March to August 2020. Prior to the onset of the trial, a situation health facility survey was conducted in three sampled government-owned dispensaries. Stratified random sampling was used to select study facilities. Firstly, government-owned dispensaries were identified in villages with no malaria control research interventions and stratified by district. Simple random sampling technique was used to select one out of 4 dispensaries in Kibiti and two dispensaries out of 5 in Rufiji district. The survey aimed to determine facilities’ readiness in screening and managing primaquine related adverse events. The survey focused on the following using a checklist; (i) human resource capacity in terms of number and qualification of health care providers, (ii) laboratory capacity in terms of available resources necessary for malaria and anaemia screening, (iii) essential supplies for managing severe acute haemolytic anaemia, and (iv) facility system and means in place for referring patients with emergence conditions. Facilities were then capacitated to manage malaria patients with uncomplicated *P. falciparum* malaria using SLD primaquine plus AL based on the findings from the needs assessment survey. Facility capacitation including familiarizing providers with PROMPT through training, pilot and adapting the tool. Facilities were being supplied with enough anti-malarials, ORS, haemoCue^®^ machine, weighing machine, stationary, Hillmen urine colour chart, and laboratory consumables necessary to manage and follow a patient treated with primaquine. Furthermore, PROMPT package documents, including patient data collection form and patient information card, were also supplied throughout the trial period. None of the research team members was present in the facilities during the trial period to support recruitment, screening, prescription, patient follow-up or site supervision. This was important to ensure the trial is implemented in a routine healthcare environment by the existing facility personnel and system without any external interference.

A tablet of 7.5 mg primaquine (Sanofi-Aventis Cyprus Limited) was used. A single dose of primaquine using PROMPT defined therapeutic dose range that is equivalent to 0.25 mg/kg primaquine base was administered; 0.5 tablet to 10–15 kg patient, 1 tablet to 16–30 kg patient, 1.5 tablets to 31–45 kg patient, and 2 tablets to patient above 45 kg [14]. A 7.5 mg base primaquine phosphate tablet was crushed and dissolved in 7.5 ml of drinking water to produce a stable 1 mg/ml solution. A sterile syringe was used to draw the assigned dose to the nearest 0.5 ml and immediately gave it to the participant in a spoon to swallow together with the sweet beverage, mainly soda and fruit punch. The approach has been described elsewhere [18]. Primaquine was administered under direct observation at the facility together with the first doses of AL (Coartem^®^, Novartis; artemether 20 mg and lumefantrine 120 mg). Patients were not screened for G6PD deficiency.

### Study design

This study employed a qualitative cross-sectional design involving in-depth interviews (IDIs) and focus group discussions (FGDs). The study was conducted in July 2020, when the trial was in its 5th month of implementation.

### Sampling

Purposive sampling was performed to achieve uniform representation of the three main FGDs of eligible patients participated in the trial including male, female, and parents or guardian with sick children. Since each study facility had only one clinician and two to three nurses, all clinicians and nurses who spend most of their time in the outpatient unit were selected to participate in the IDIs in each facility. All the study facilities did not have a laboratory or pharmacist attendant.

### Data collection

A total of six (three clinicians, three nurses) IDIs was conducted with the healthcare providers (HCP) from all three facilities. IDIs collected information on provider’s perceptions and experience in patient screening, PROMPT data collection form, PROMPT patient information card, primaquine prescription and dosage, patient follow-up, and treatment safety concerns. Six FGDs were conducted with patients (with 24 males and 27 females) treated with SLD primaquine involving three groups; adult men, adult women, and parents or guardians with malaria-treated young children. The breakdown for the six FGDs is: 2 with males, 2 with females, and 2 with parents/guardians with sick children. Each FGD had 8 to 9 participants and explored patients’ perception of overall management experience from providers, day 7 follow-up, treatment safety concern, and outcome. The IDI and FGDs were conducted using a topic guide that had been pilot-tested. Two experienced qualitative researchers conducted IDIs and FGDs in Swahili and in a private place to ensure confidentiality. All the IDIs and FGDs were audio recorded and transcribed verbatim and later translated into English.

### Data analysis

Thematic analysis was done with the aid of NVIVO qualitative analysis software. Transcripts were imported into NVivo, and coding was done by two researchers involved in the data collection. Transcripts were initially read and a priori and emerging codes were developed. Two researchers, including the first author, examined the coded data and developed it into themes. Hypotheses were generated through repeated reading of themes.

### Ethical considerations

This study was conducted in accordance with the Declaration of Helsinki, ICH Good Clinical Practice, and local regulatory requirements from the Tanzania Medicines and Medical Devices Authority (TMDA). The study is registered with the Pan African Clinical Trial Registry (Number PACTR202007653561223). Ethical approval was granted by the Ifakara Health Institute (IHI) ethical review board and the Tanzania National Research Ethics Committee (Number NIMR/HQ/R.8a/Vol.IX/3114). Voluntary written informed consent was obtained from all study participants. Confidentiality of the data obtained from participants was strictly secured and maintained.

## Results

The median age of study participants was 35 (ranging from 27 to 46) years. Providers had on average, had 6 years of experience providing health care and had worked in the study area for at least 3 years. Among the three clinicians, two were males and one female. All clinicians had the academic qualification level of medical assistants, referred to as clinical officers in Tanzania. All the nurses were females with the professional title of Enrolled Nurses. At the time of the interview, each facility had already treated at least 150 patients with SLD primaquine co-administered with AL.

The age of the FGD participants ranged from 19 to 44 years, and their education level ranged from no school to ordinary secondary school level. All participants were residents of the study area and had a prior experience being treated for malaria with AL only.

### Patient screening experience

All participating HCP supported the importance of screening patients for the recommended treatment eligibility criteria, starting with a detailed patient medical history and conducting a physical examination and laboratory tests. This is also part of their medical training to screen all patients before treatment and not only for suspected malaria cases. More than two-thirds of the providers mentioned that screening patients for SLD primaquine as outlined in the PROMPT is clear to follow and guides them better towards the appropriate management, particularly to some criteria that can easily be overlooked and missed.

*“The screening information in the guide helps me to make sure I have assessed all patient’s eligibility criteria for this new malaria treatment otherwise, you can easily miss important medical conditions such as an allergy to other malaria drugs or even pregnancy”*. IDI, Nurse aged 46

*“Routine screening for anaemia has helped us a lot to diagnosed malaria patients with severe anaemia because previously, we did not often measure Hb when we see a child is stable. Now we are surprised to see a child with Hb below 4 g/dl and is stable and walking”*. IDI, Clinical Officer aged 35

HCP mentioned some challenges regarding patient screening. About half of the providers said that they spend more time attending to a patient because of going through the eligibility criteria and screening for anaemia even though they were using a rapid anaemia screening machine (HemoCue^®^ machine). Some providers were also concerned about the sustainability of routine screening for anaemia because of limited supplies to support such an exercise beyond the programme funding period.

One provider mentioned that the workload at public health was already too much:

*“Explaining to the patient about this new treatment and anaemia screening to all before treatment is somehow demanding in our facility because I am the only clinician here together with two nurses who spend most of the time in reproductive and child health (RCH) clinic and labour room, and no laboratory officer…., so you can imagine the workload I have, measuring Hb to all patients receiving primaquine and at the same time managing other cases as well as facility administrative duties”*. IDI, Clinical Officer aged 35

In reference to the stockout challenge, one provider expressed her scepticism in the following:

*“…… I’m not sure about how this be sustained considering the stock out of supplies, particularly the trips used in the Hemocue machine for measuring Hb is a chronic problem in our district. A facility may take up to two months or more without measuring Hb, forcing us to refer patients to other facilities only to check Hb”*. IDI, Nurse aged 34

The majority of the patients mentioned the providers took more time exploring their medical history when comparing with their previous experience in the same facility. They felt more valued by the clinician by showing more concern about the presenting illness and even taking blood for anaemia screening whenever they are found to have malaria.

*“…… in the past, doctors appeared to be rushing in order to clear the number of patients waiting for his service … he may just ask you few questions about how you feel and immediately tells you to go and check for malaria. At times you even hesitate to ask him all your questions, assuming that you might waste his time….. This time is different; he asks you different questions and even wants to know whether you are pregnant while you just came for malaria treatment”*. FGD, female patients

### Patient treatment information

HCP provided the information on primaquine treatment and possible adverse events, including assessing their urine colour changes at home, having the Hillmen Urine Colour Chart for reference. They were also insisted on returning to the clinic on day 7 to follow up with all patients. They also reported having given all patients a PROMPT patient information card, a sheet of paper with about 70 words that mention the treatment received, possible primaquine side effects, the date for day 7 follow visit and the provider’s cellphone number. Nearly all providers mentioned patients are happy with the information card provided, especially by including the provider’s phone number.

*"Patients are happy with the sheet we are providing to them because it has our phone numbers so as they can call and ask questions when they are at home or even sometimes when the facility is closed”*. IDI, Clinical Officer aged 38

HCP reported that they frequently received regular calls from patients and responded to their questions, including matters unrelated to malaria.

*“Our phone numbers provided in the card makes it easier for the patients to reach us whenever they see something is wrong with them….. I remember the last call came from a patient complaining of having dark urine, and I told him to come back immediately in the morning for a checkup”*. IDI, Clinical Officer aged 35

Patients are happy with the information card because apart from the providers’ phone number provided, they also mentioned that the information card reminds them of the adverse events related to the primaquine use. However, the information card was not as useful to illiterate patients, and most depended on literate family members to read for them.

*I was provided with a sheet explaining the treatment, which I think is something good, but I couldn't read it, and no one at home could help me”*. FGD, female patients

Many patients also attested to the good service from the HCP. They reported that the information provided was clear and useful even for those who could not read or write.

*“…… as you can see most of us can’t read this information sheet at home due to different reasons but I think the information explained by the doctor in the facility was clear and the good thing in this sheet there is doctor’s phone number, and she said to call her at any time when we see something is going wrong”*. FGD, male patients

HCPs also mentioned that despite being aware of most of their patients being illiterate, they still provided information cards to all treated patients as per the PROMPT protocol.

*“Many people in our community don't know how to read and write, but we still give them the form and advise to ask someone at home to read for them for more understanding”*. HCP in IDI 03

Contrary to the broader positive view regarding providing treatment information to the patients as indicated in the PROMPT, there were other opinions whereby providers found it challenging. They feel some patients do not understand the message clearly when explaining to them in the clinic. Some take mixed messages to the community about the treatment leading to misperception of the treatment.

*“Level of understanding of patients is very low. Some don't understand and at some point, they send different message messages to the community about primaquine prescribed”*. IDI, Nurse aged 34

Nearly half of the providers mentioned limited time to explain to each patient clearly about the treatment and responding to patients queries as a key challenge.

“*Sometimes it is hard to explain in detail everything to the patient about primaquine due to a big number of patients who are waiting to be attended, and some are in critical condition hence, forces us to summarize the information on the key safety issues*”. IDI, Clinical Officer aged 32

### Primaquine prescription and administration

All the providers mentioned the primaquine dosage chart from PROMPT defined therapeutic dose range and this is constructive as it guides them to choose the appropriate dose based on the patient’s weight. The challenge mentioned was when there is a need to crush a tablet and come with the proper amount, particularly for patients with the age range of 10–15 kg (require 0.5 tablet of primaquine 7.5 mg) and 31–24 kg (require 1.5 tablets of primaquine 7.5 mg). All providers talked about the challenge related to the administration of primaquine by dissolving first the tablet of 7.5 mg in a small glass tube with 7.5 ml clean water. After that, draw with a syringe to get the required patient dose as instructed in study training for treating malaria with SLD + ACT. Nearly all providers were also concerned about the bitter taste of dissolved primaquine and thus were administering it to the patient together with a sweet beverage to mask the bitterness. This was thought to subject a patient to unnecessary cost in the absence of support from the study. All the providers recommended the need to have a new primaquine formulation that requires no crushing or dissolving the tablet.

*“It is very challenging for us to dissolve first the tablet in water to estimate the recommended patient dose. This is something that is new to us and very difficult in the actual practice of our environment….. we think it is an unnecessary additional cost to the patient by taking soda together with dissolved primaquine tablet so as not to feel the bitterness”. *IDI, Nurse aged 34

*“….good to have dose-specific tablets for patients according to their age and weight like other treatment and not to crush the tablet that is very small than piriton (chlorphenamine tablet which is considered to be the smallest tablet by many providers)”. *IDI, Nurse aged 27

However, patients including parents and guardians with young children, mentioned that it was not a problem taking a tablet dissolved in water. The sweet beverage provided for swallowing primaquine made the experience of swallowing the medicine desirable among children.

*“The medicine was ok and my child was fine with the taste, I think it was because of the soda used to diluting the medicine… sometimes at home, they are asking if we can go again to the hospital and take the malaria medicine”*. FGD, parents/guardian

### Patient follow-up experience

Most of the patients supported the importance of follow up in the facility after treatment to ensure that they were progressing in good health. However, they were concerned about the transportation challenges when coming to the facility for the scheduled follow up visit. Reflecting on access barriers and transport costs for a return visit, a patient reported:

*“The challenge of coming back on day seven after a treatment has transportation challenges to some of us who are staying far from this facility… we use a lot of money in the commuter bus and bodaboda (motorbike taxi) to reach to this facility”*. FGD, female patients

A second patient talked about access in the following:

*“Many of us are staying far from the facility and the road is poorly accessible especially during the rainy season that’s why some of our friends failed to come back… but I assure you if you want all of us to come back as scheduled, then you should consider reimbursing our transportation expenses”*. FGD, male patients

All HCP supported the importance of patients returning on day 7 for the follow-up. Determining the haemoglobin status post-treatment was the main reason for the latter. A big drop in haemoglobin level was observed among most patients, albeit no one mentioned the incidence of significant haemoglobin drop, leading to severe anaemia.

*“….this is very important to follow patients so as to be sure of their Hb (haemoglobin) status after treatment because of what we observed… I can say most patients presented with Hb drop of about 1 to 3 units from the original value”*. IDI, Clinical Officer aged 38

HCPs were also concerned with patients transportation challenges, responsible to some patients failing to return to the clinic for follow up. Failure to return was perceived to be attributed to patient behaviour. When the patient felt better after completing the medication, they were least motivated to see a clinician:

*“Some patients don’t come on day 7 as scheduled and instead may come on day 10 or not at all simply because of transportation challenges to access the health facility which is contributed by long distance to the facility and poor road condition, having mud and flood”*. IDI, Clinical Officer aged 32

### Data collection form

More than two-thirds of the providers found the PROMPT data collection form useful as it helped them ensure that they had noted down all the necessary information required in making a treatment decision. They were not used to only one person completing a similar form in a specific fixed order. They mentioned the latter as one of the reasons for a prolonged time in attending to malaria patients. They suggested that the form should be shortened and that this should be incorporated with the existing medical information system.

*“We are not used to documenting patients information in detail, covering all issues about the treatment by following a specific order, step by step, and then provide our phone numbers to all patients attended. Initially, it was a big burden in terms of time to make sure we are not making mistakes, but as time goes on, we improved the speed and accuracy”* [IDI, Nurse aged 34]

### Perceived primaquine safety and outcome

All HCP reported no cases of severe adverse events such as death, severe anaemia or case referred for blood transfusion or any reason for using SLD primaquine in the management of uncomplicated malaria. The treatment safety concern mentioned by nearly all providers was the drop of haemoglobin level in many patients observed on day 7 of follow up but not to the level that may require a blood transfusion. Two providers each mentioned to have seen the patient coming back complaining of dark urine, and after 2 days, the urine was clear. HCPs mentioned managing this condition by prescribing oral rehydration therapy (ORS) and was used at home for 2 days. None of the providers received a patient with serious complaints linked to primaquine, such as stomachache, nausea or vomiting.

*“I only have experience of one patient presented with dark urine after using the treatment, we told him to come back on the following day and confirmed his urine was dark, but we did not check his Hb because day 0 Hb was around 12 points and something (12 mg/dl). We then gave him 4 sachets of ORS (for 4 l) and encouraged him to use them at home; after two days, he called us again and was happily informing us he is doing ok”. *IDI, Nurse aged 46

Nearly half of the providers mentioned the treatment has helped reduce the turn-up of patients to the facility due to recurrence of malaria. This was observed since they started including SLD primaquine in treating patients with ACT.

*“The treatment has reduced a big number of patients returning to our facility with malaria in a few days after completing malaria treatment. Now it can take several months for patients to return to the facility for malaria treatment… I can say this new medicine has also reduced the number of malaria attended cases”*. [IDI, Clinical Officer aged 35]

A similar experience was also mentioned in one FGD with parents and guardians:

*… every month, my children were suffering from malaria, but since they started getting this treatment, at least a month has now passed without having malaria in my family”*. FGD, parents/guardian.

## Discussion

The study has shown that a single-low dose primaquine regimen and pharmacovigilance monitoring tool among healthcare providers and patients in a real-world environment is feasible and acceptable. Although some providers considered it time-consuming, patient screening procedures and treatment information were perceived to be good and clear to both providers and patients. Primaquine administration had practical challenges in some patients who needed to crush and dissolve first the tablet to get an appropriate dose. According to the schedule, returning to the clinic for treatment follow-up on day 7 was challenging for patients due to varied reasons.

The treatment was generally perceived as safe by both patients and providers with no reported severe adverse events. Providers were concerned about the haemoglobin level drop observed on the scheduled follow-up visit on day 7. Screening for the treatment eligibility criteria as per the PROMPT that includes assessment of the specific patient safety condition through medical history, clinical and laboratory investigations assisted the clinician in identifying life-threatening conditions, particularly severe anaemia. Routine laboratory screening for haemoglobin level is often ignored in patients diagnosed with malaria in many low-income countries, reportedly due to resources constraints [19]. This may suggest the need to use other cheap alternatives for assessing anaemia, such as the WHO Haemoglobin colour card in order to sustain anaemia screening. However, the WHO Haemoglobin colour card is subjected to errors [20]. The present study demonstrated the acceptance of providers to screen all patients for anaemia before treatment. Children with pre-existing anaemia are at risk of severe adverse outcomes when co-infected with malaria or as a secondary consequence to acute haemolytic anaemia, risk in those patients with G6PD deficiency [21]. In another paper to be submitted shows that under-five children included in the study had a mean haemoglobin of 10.3 g/dl. Using the PROMPT, patients with haemoglobin below 7 g/dl were excluded from receiving SLD primaquine. This may explain why no severe adverse event was reported during the trial period, considering the prevalence of anaemia among children in the study area is above 40% [22].

No study facility could examine blood slides to determine parasitaemia level or type of malaria parasite because they had no laboratory technician. Therefore, malaria diagnosis in the present study was based on RDT, the test that is considered more reliable and saves time in resource-limited countries, countries mostly challenged by having unskilled personnel [23]. The current study design did not screen for G6PD or urine colour before treatment as they are not primary screening requirements in the PROMPT. However, none of the providers perceived the two screening tests during the baseline would affect the treatment outcome.

Good providers patients interaction, including understanding patient’s medical condition, provides the patient with relevant detailed information about the treatment, and importance for patients to return to the clinic for follow-up, as emphasized in the PROMPT, have been reported to improve the quality of care and adherence to treatment [24]. Patients in the present study were happy to see providers take extra time explaining to them about the treatment, which is different from their previous experience. The patient–provider relationship should therefore be promoted to ensure better treatment outcomes.

The limited number of health providers in many resource-limited countries, including Tanzania, are overwhelmed with many patients in the clinic. This has been reported to affect the quality of care in these countries [25]. Time constraints to provide enough information to patients about primaquine besides performing all treatment screening procedures was reported to be a big burden by nearly all providers in the study. With the new malaria treatment regimen, advocacy to the community about the treatment should be emphasized to improve the client’s knowledge in advance before attending the facility for medical attention. This may reduce the time needed for the provider to explain about the treatment. Promoting advocacy to the community and opening new treatment dialogue has been demonstrated to minimize mixed messages about the treatment and improve treatment uptake and adherence [26].

Most patients appreciated the PROMPT patient information sheet provided by healthcare providers on the first day of treatment, though some could not read and write. The latter should not ignore the value of supplement information material to patients because multiple information channels have reported improving understanding and knowledge of a particular subject matter [27]. Disclosing the phone number of a provider in the PROMPT patient information sheet aimed to facilitate patient-provider communication, mainly when a patient is far from the facility and needs immediate medical attention or advice. This should be encouraged considering optimal patient–provider communication has been reported elsewhere in the outpatient clinic setting to be critical for safety and effective patient care [28]. However, phone calls from patients were considered a burden and irrelevant to some providers because they were not related to the treatment. This can be addressed by always encouraging patients not to call providers for non-medical reasons.

PROMPT defined therapeutic dose range is a novel idea from MEI research work that aims to simplify primaquine dosing regimen and minimize errors that may affect drug safety and efficacy to the patients [14]. Most providers reported the usefulness and simplicity of the therapeutic dose range guide in determining a patient’s dose. A simplified dosing chart for treating a patient has been reported to reduce human errors [29]. Therefore, there is a need to encourage using the PROMPT defined therapeutic dose range guide in treating malaria with SLD primaquine, especially in the resource-limited countries where over-the-counter prescription among non-medical personnel is common [30, 31].

Administration of primaquine was reported to be challenging by all providers, particularly where there is a need to crush the tablet first in order to get the appropriate dose based on the patient’s body weight. This is due to a small tablet size of around 6 mm in diameter, making it almost impossible to split it evenly. Unfortunately, the lack of the WHO prequalification primaquine tablet with lower strength has been reported to be a challenge by manufacturers due to limitations related to formulation processes [13]. A possible way forward for consideration is to develop a dose band with commonly used ACT. SANOFI is currently working on developing accessible primaquine dispersible tablets for children [32]. Allowing providers to continue administering SLD primaquine by first crushing and dissolving the tablet, particularly in the paediatric population, may increase the likelihood of administering a wrong dose, as demonstrated elsewhere [33]. The bitter taste of a dissolved primaquine tablet may also discourage the patient from taking it. A good acceptance observed among children to take a crushed primaquine tablet may be explained by mixing it with a sweet beverage.

Several studies have shown follow-up on patients after or through the treatment process to improve patient satisfaction, medical safety and ensure the health-related quality of life [34, 35]. Most patients also reported the importance of returning to the clinic for medical follow-up and screened for haemoglobin levels regardless of their perceived good clinical progress. This should be encouraged when rolling out SLD primaquine because it helps to detect any drug-related adverse events, particularly acute haemolytic anaemia [14]. However, poor access to the health facility and transport costs were reported by patients to be the key factors responsible for failing to return for the scheduled follow-up visit. Transport cost has also been reported to be the main challenge of patients lost to follow-up for their routine treatment and care in other sub-Saharan African countries [36, 37]. Therefore, there is a need to explore interventions that will facilitate patients to turn up to their scheduled follow-up visits. Interventions such as making phone calls or sending reminder phone messages may be effective considering mobile phone coverage is good in many sub-Saharan Africa countries [38]. Sending reminders has proved to improve the likelihood of the patient keeping the medical appointment [39].

There was no severe adverse outcome observed or reported by either providers or patients. This agrees with the rich available information about the safety of SLD primaquine in treating *P. falciparum* malaria [7–9]. Providers were mainly concerned with the haemoglobin level drop observed at day 7 of follow-up compared with the baseline haemoglobin level. A few days after malaria treatment, the haemoglobin level drop could be mainly due to haemolysis of parasitized red blood cells regardless of the patient’s G6PD status [40]. Whether the observed day 7 haemoglobin drop was significantly associated with the G6PD status of patients will be presented separately in the quantitative paper.

## Conclusion

This study provided findings on acceptability and the safety monitoring of SLD primaquine in routine healthcare practice in rural and the lowest level of health facility delivery in low-income countries. Acceptance of the SLD primaquine and the use of PROMPT among healthcare providers in managing malaria is encouraging. The challenges observed in the present study calls upon the need to validate and customize the primaquine safety monitoring tool to a local context by working with the national malaria control programme before SLD roll-out.

## Data Availability

Not applicable.

## Abbreviations

ACT: Artemisinin-based combination therapy
AL: Artemether–lumefantrine
FGD: Focus group discussions
G6PD: Glucose-6-phosphate dehydrogenase deficiency
IDIs: In-depth interviews
IHI: Ifakara Health Institute
MEI: Malaria Elimination Initiative
NMCP: National Malaria Control Program
NIMR: National Institute for Medical Research
PROMPT: Primaquine roll out monitoring pharmacovigilance tool
TMDA: Tanzania Medicines and Medical Devices Authority
WHO: World Health Organization
